# A case report of post-acute COVID-19 encephalopathy

**DOI:** 10.1192/j.eurpsy.2023.1645

**Published:** 2023-07-19

**Authors:** S. Cekerinac, D. Sekulic, B. Batinic

**Affiliations:** 1 Department of Psychiatry, General Hospital, Sremska Mitrovica; 2 Department of Psychology, University of Belgrade, Faculty of Philosophy; 3 Clinic of Psychiatry, University Clinical Centre of Belgrade, Belgrade, Serbia

## Abstract

**Introduction:**

Following the protracted duration of the coronavirus pandemic, the Serbian health system now faces a period of mid- and long-term health consequences in patients that have recovered from the acute phases of infection.

**Objectives:**

A 57-year-old woman presented at a psychiatric examination complaining of forgetfulness, listlessness, fatigue, insomnia, low mood, and decreased efficacy in daily activities, two months after infection with the SARS-CoV-2 virus. The clinical picture of acute COVID-19 infection was accompanied by an elevated body temperature, a cough, an increases of CRP, and X-ray verified bilateral pneumonia with band-like speckled shadows of milk glass density. Before infection, she was vaccinated with 3 doses of the Sinopharm Covid-19 vaccine.

**Methods:**

The following examinations were made: MRI of endocranium, HDRS, laboratory examination, and neuropsychological testing.

**Results:**

MRI of endocranium (figure 1): extensively cortico-subcortical lesions extensively within both cerebral hemispheres, dominantly in the temporo-insular regions, in association with partial parenchymal defects and a high degree of atrophy - the overall morphology corresponds to chronic encephalopathy, which is of non-specific morphology; HDRS score of 24; elevated serum levels of IgM, IgG, albumin in serum 7.05 (35-55), albumin in cerebrospinal fluid 812.0 (0-35), albumin index 115.8 (<9.0); IEF: oligoclonal bands in CSF and serum; neuropsychological testing: decrease in general mental activity and visuoperceptive and visuospatial ability. Due to the temporal connection between infection with the SARS-CoV-2 virus and presented symptoms, the patient was diagnosed with post-acute COVID-19 encephalopathy.

**Image:**

**Figure:**
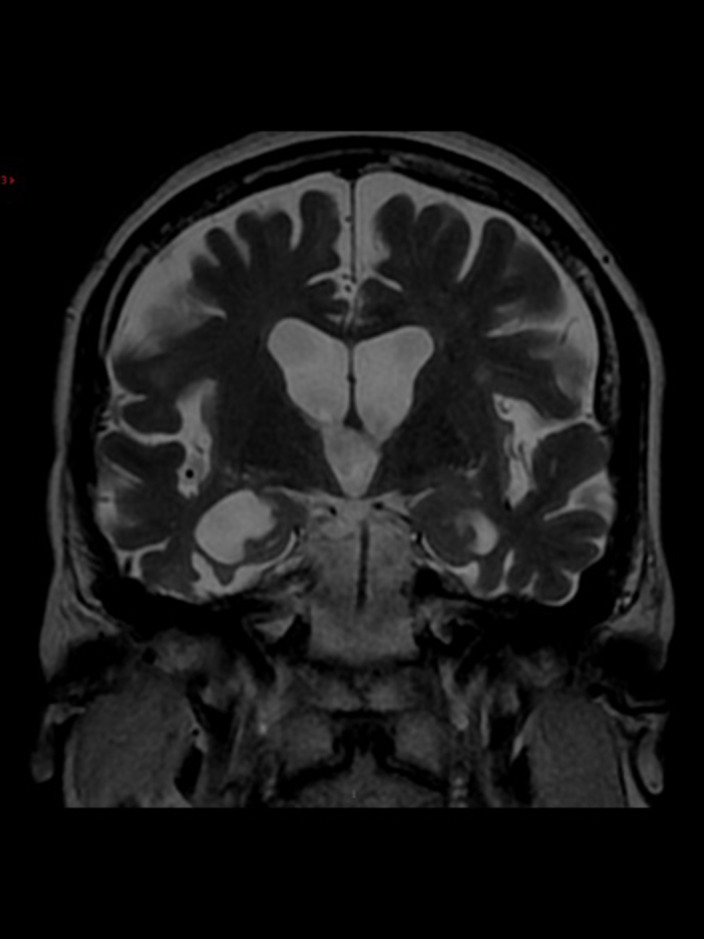

**Conclusions:**

A meticulous follow-up post-acute SARS-CoV-2 infection monitoring and care could decrease mortality and prevent debilitating neurological and other burdens, especially in risk groups.

**Disclosure of Interest:**

None Declared

